# Specification-driven acceptance criteria for validation of biopharmaceutical processes

**DOI:** 10.3389/fbioe.2022.1010583

**Published:** 2022-09-23

**Authors:** Lukas Marschall, Christopher Taylor, Thomas Zahel, Marco Kunzelmann, Alexander Wiedenmann, Beate Presser, Joey Studts, Christoph Herwig

**Affiliations:** ^1^ Körber Pharma Software, Vienna, Austria; ^2^ TU Wien, Institute for Chemical, Environmental and Bioscience Engineering, Vienna, Austria; ^3^ Boehringer Ingelheim Pharma GmbH & Co, Biberach an der Riss, Germany

**Keywords:** integrated process model, statistical modelling, bioprocess, control strategy, acceptance criteria, specification limits, process validation, DOE

## Abstract

Intermediate acceptance criteria are the foundation for developing control strategies in process validation stage 1 in the pharmaceutical industry. At drug substance or product level such intermediate acceptance criteria for quality are available and referred to as specification limits. However, it often remains a challenge to define acceptance criteria for intermediate process steps. Available guidelines underpin the importance of intermediate acceptance criteria, because they are an integral part for setting up a control strategy for the manufacturing process. The guidelines recommend to base the definition of acceptance criteria on the entirety of process knowledge. Nevertheless, the guidelines remain unclear on how to derive such limits. Within this contribution we aim to present a sound data science methodology for the definition of intermediate acceptance criteria by putting the guidelines recommendations into practice (ICH Q6B, 1999). By using an integrated process model approach, we leverage manufacturing data and experimental data from small scale to derive intermediate acceptance criteria. The novelty of this approach is that the acceptance criteria are based on pre-defined out-of-specification probabilities, while also considering manufacturing variability in process parameters. In a case study we compare this methodology to a conventional +/- 3 standard deviations (3SD) approach and demonstrate that the presented methodology is superior to conventional approaches and provides a solid line of reasoning for justifying them in audits and regulatory submission.

## 1 Introduction

Process Validation for the pharmaceutical industry is “the collection and evaluation of data, from the process design stage throughout production, which establishes scientific evidence that a process is capable of consistently delivering quality products.” (FDA, 2011). This involves a series of activities taking place over the life cycle of the product and process. The goal of process validation is to set-up and maintain a control strategy that enables the process to continuously deliver product quality. This desired quality is defined by the quality target profile (QTPP) of a product (ICH Q8, 2009, S. 8) and acceptable quality limits are defined by drug substance and drug product specification limits. The final gate keeper for the market release of product from a manufacturing process are the drug product specification limits for each of the individual attributes of the QTPP, referred to as Critical Quality Attributes (CQAs).

Amongst other goals, a control strategy aims to control 3 types of parameters: process parameters (CPPs), material attributes (CMAs) and the quality attributes themselves (Burdick et al., 2017). In process design, depicting phase 1 of process validation, process parameters and material attributes are assessed and investigated (FDA, 2011). Their impact on product quality and process performance is studied and quantified in experiments. Dependent on the observed effects on product quality, appropriate control ranges are defined for process parameters and quality attributes. Most commonly, each process step (or unit operation) is investigated individually. However, to define the control ranges of CPPs and CMAs, it is important to know which quality attribute levels are acceptable at each process step (Jiang et al., 2010).

**FIGURE 1:**
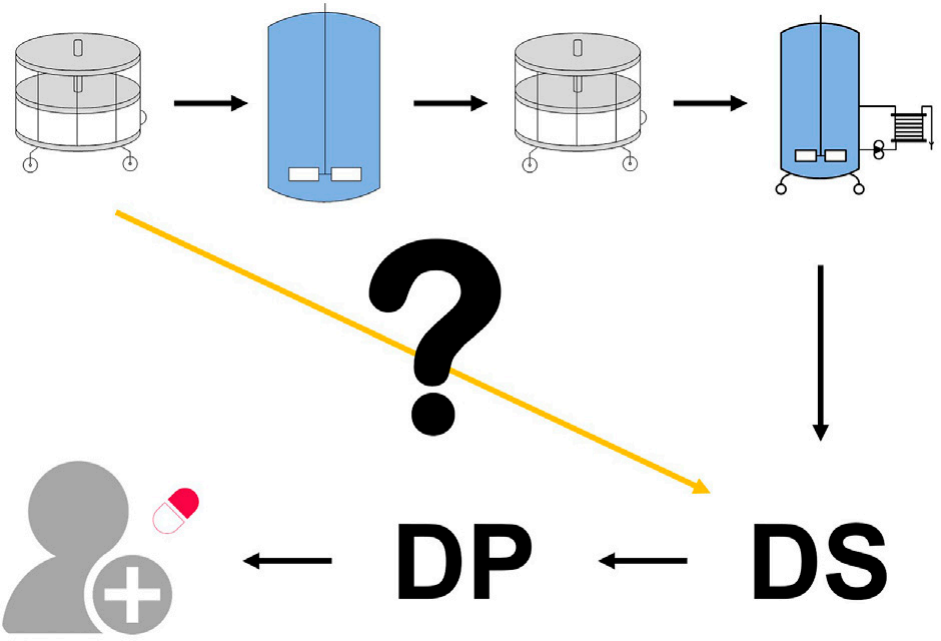
*Drug substance specifications define the quality limits that must be achieved before product release. However, these are only assessed at the final unit operation. There is no standardized methodology for assessing the first unit operation’s impact on the drug substance (yellow line) nor is there a standardized method for linking any derived intermediate acceptance criteria with the final drug substance specifications*.

In ICH Q6B, an acceptance criterion is defined as *“An internal (in-house) value used to assess the consistency of the process at less critical steps.”* (ICH Q6B, 1999, S. 6). Within this contribution, we focus on acceptance criteria for CQAs at intermediate process steps (Figure 1). Hence, we refer to these limits as intermediate acceptance criteria. They describe which quality levels each unit operation has to deliver, whereas the drug substance or product specification limits describe, which quality levels the process has to ultimately deliver before product release.

Without knowing which quality levels are acceptable at each process step, it is difficult to set up control ranges for CPPs and CMAs at the respective process steps. As managing the risk to quality is regarded to be the ultimate goal (ICH, 2005), deriving these limits is crucial for the success of a process validation project. EMA-FDA, also requires acceptance criteria for CPPs and CQAs to be part of the process validation protocol (European Commission, 2015). Per these guidelines, the acceptance criteria should be based on development data or documented process knowledge. If the measurement of quality attributes in the process are part of the control strategy (as in-process controls), intermediate acceptance criteria (iACs) are required and solid rationales should be provided for their establishment.

There are currently multiple methods to derive iACs for quality attributes.

One solution to define iACs is by performing wet-lab spiking studies. This is an approach commonly applied in virus clearance studies (Darling, 1993). EMA also explicitly mentions spiking experiments to demonstrate the clearance capacity of downstream unit operations for host-cell relate impurities (EMA/CHMP/BWP/187338/2014, 2016). However, finding the correct spike material is difficult, as care has to be taken that the sample matrix is not completely altered by other components contained in the spiking material and correctly represents the material in the naturally occurring process.

Acceptance criteria may also be calculated based on data collected at set-point conditions. They can be calculated by applying +/- 3 standard deviations (3SD) of the existing data, or statistical intervals based on an assumed underlying distribution (e.g. tolerance intervals). These approaches do not account for variability around process parameters and don’t provide a linkage to drug substance specifications (Seely et al., 2003; Orchard, 2006; Wang et al., 2007). Moreover, both approaches heavily rely on the observed variance. Higher variation leads to wider acceptance criteria and lower variation to tighter limits. Both approaches reward poor process control and punish good process control. Moreover, the mentioned methods are focused on individual unit operations only.

Another approach linking knowledge across multiple unit operations is described by Montes (Montes, 2012). They compare methods to estimate the to-be-expected variance at each process step. One of the discussed approaches is to apply variance transmission. The variance for e.g. process step 3 is calculated by applying error propagation using the known regression models for process steps 1 to 3. The estimated variance is used to calculate tolerance intervals. The worst case side of the tolerance interval (in the case of a two-sided interval) is then used as acceptance criteria for the respective step. This approach leverages the knowledge of known functional relationships. The defined acceptance criteria give information on the possible worst case of a process at the observed variance. However, they don’t give any information how likely it is to meet drug substance criteria.

Monte Carlo approaches have been applied to the definition of specification limits (Burdick et al., 2017). Burdick et al. used the approach to calculate the final distribution of a drug product quality attribute after several storage steps and suggested to use the calculated distribution to derive specification limits.

Ideally, iACs share the following characteristics:

•) iACs should provide a link to drug substance or product limits: the likelihood or probability of meeting drug substance specifications while staying within the intermediate acceptance criteria.
•) iAC derivation should consider the uncertainty around process parameters and material attributes

Within this contribution, we build upon the concept on integrated process modelling as described by Zahel et al. (Zahel et al., 2017). In an integrated process model (IPM), each unit operation is described by a multilinear regression model where the performance (clearance or purification capability) is the dependent variable and the input of the previous unit operation as well as the process parameters act as independent variables. These models are built with large scale data from manufacturing and small scale data from process characterization studies.

The models are concatenated by using the predicted output of a unit operation as input for the subsequent unit operation. Using Monte Carlo simulation, random variability caused by process parameters can be incorporated into the modeled process (Zahel et al., 2017). IPMs can be used to predict the out-of-specification probability for a given set of process parameter set-points. Another application is to set up a control strategy for process parameters by defining proven acceptable ranges (Taylor et al., 2021).

**FIGURE 2:**
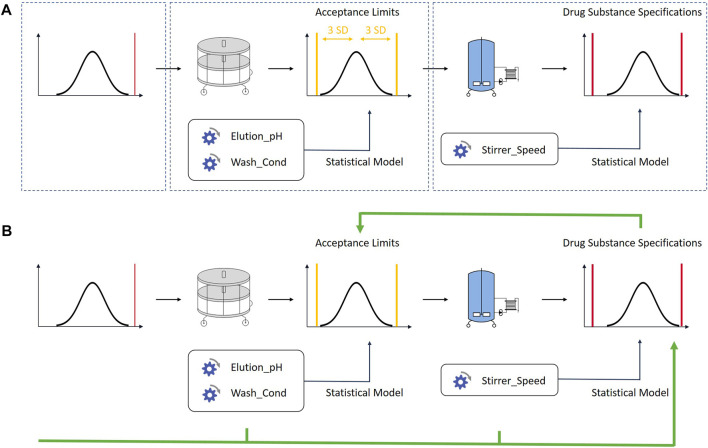
**(A)** *One of the current best practices approaches. Control strategies are set up for each unit operation individually. The defined acceptance criteria are not linked to drug substance specifications*. **(B)** *The methodology described in this contribution links iACs to drug substance specification limits using an integrated process modelling approach. Each unit operation is described by one ore multiple multilinear regression models built with large scale data from manufacturing and small scale data from process characterization studies. The models are concatenated by using the predicted output of a unit operation as input for the subsequent unit operation. By doing so, acceptance criteria can be based on drug substance limits. The in-silico linking of unit operations has also been discussed in other contributions* (Montes, 2012; Burdick et al., 2017)

Within this contribution, we aim to derive iACs that ensure a pre-defined out-of-specification probability. These specification-driven ranges enable the set up of a control strategy that prevents failed batches at highest possible manufacturing flexibility. The novelty of this approach is that the acceptance criteria are based on pre-defined out-of-specification probabilities, while also considering manufacturing variability in process parameters (Figure 2).

The manuscript is structured in two parts. First the developed method is described. In a second step the developed method is applied to a real world case study and compared to a conventional approach.

## 2 Methods and materials

### 2.1 Candidate process for case study

For the case study, a monoclonal antibody (mAb) production process in mammalian cell culture was provided by Boehringer Ingelheim in Biberach, Germany. The model depicts the downstream process segment of the drug substance manufacturing process.

The downstream process consists of 9 unit operations. The first step is the pool of the harvested fermentation broth (UO 1), the second step is a chromatographic capture step (UO 2), followed by a viral inactivation (UO 3), depth filtration (UO 4), two chromatographic steps (UO 5, UO 6), a viral filtration (UO 7), another chromatographic step (UO 8) and ultra-and diafiltration (UO 9).

Three quality attributes defined as CQAs were modelled. One product-related impurity (UP-SEC Aggregates) and one host-related impurity (HCP ELISA) that need to be cleared by the downstream process and one parameter, purity (UP-SEC Monomer), that should be increased.

### 2.2 Data for the integrated process model

For the capture chromatography, the virus inactivation and the anion exchange chromatography one-factor-at-a-time (OFAT) studies were performed. For the cation exchange chromatography 2 factors were investigated in a design of experiments (DoE) approach. One factor was varied in 5 levels and the second factor in 3 levels. One center-point was performed. The design is able to resolve main effects and quadratic effects. For the hydrophobic interaction chromatography 3 factors were investigated in a face-centered central composite design with 3 center points. The design is able to resolve main effects, two-factor interactions and quadratic effects. All experimental studies were performed in small scale.

The available data for each unit operation is summarized in Table 1.

**TABLE 1:** Available data sets, process parameters, and monitored critical quality attributes (CQAs) for each unit operation included in the IPM.

Unit Operation	Available datasets	PPs varied in DoEs	Monitored CQAs
Harvest	10 Manufacturing Runs (2 kl)	Load Pool Temperature	HCP ELISA
Capture Chromatography	5 OFAT Runs (3L), 10 Manufacturing Runs (2 kl)		HCP ELISA, UP-SEC Aggregates, UP-SEC Monomer
Virus Inactivation	5 OFAT Runs (3L), 10 Manufacturing Runs (2 kl)	Stirrer Speed	HCP ELISA, UP-SEC Aggregates, UP-SEC Monomer
Depth Filtration	10 Manufacturing Runs (2 kl)	-	HCP ELISA, UP-SEC Aggregates, UP-SEC Monomer
Anion Exchange (AEX) Chromatography	4 OFAT Runs (3L), 10 Manufacturing Runs (2 kl)	Equilibration_pH	HCP ELISA, UP-SEC Aggregates, UP-SEC Monomer
Cation Exchange (CEX)Chromatography	11 DoE Runs (3L), 10 Manufacturing Runs (2 kl)	Elutions buffer Cond, Elutions buffer pH	HCP ELISA, UP-SEC Aggregates, UP-SEC Monomer
Viral Filtration	10 Manufacturing Runs (2 kl)	-	UP-SEC Aggregates, UP-SEC Monomer
Hydrophobic Interaction (HIC) Chromatography	17 DoE Runs (3L), 10 Manufacturing Runs (2 kl)	Loading Pool_pH, Loading Pool_Conductivity, Loading Pool_Temperature	UP-SEC Aggregates, UP-SEC Monomer
Ultra- and Diafiltration	10 Manufacturing Runs (2 kl)	-	UP-SEC Aggregates, UP-SEC Monomer

### 2.3 Calculation of performance indicators

Clearance parameters were calculated for each impurity (i) according to Eq. 1, where i is the specific impurity concentration, i.e. units per mg product, in load or pool of the respective process step.

$$SC_{i\;}={Specific\;Clearance}_{i}=\frac{i_{load}}{i_{pool}}$$

For product quantity and purity attributes, yields were calculated according to Eq. 2, where i is the product amount or percentage of desired isoform in load or pool of the respective process step.

$${Y_{i}=Yield}_{i}=\frac{i_{pool}}{i_{load}}$$

Eq. 2

### 2.4 Modelling the individual unit operations

Ordinary least squares (OLS) regression was used for statistical analysis. Scale was treated as fixed effect. As dependent variables, clearances and yields were used. A clearance represents the ratio of two assumed-to-be normally distributed random variables. Therefore, a clearance is not normally distributed. After analysis of the residuals it was decided to log-transformed the responses prior to modelling. All independent variables were scaled between -1 and 1 according to Goos and Jones (Goos & Jones, 2011). The independent variable (response) was neither scaled nor centered. For the analysis of the DoEs, a best subset variable selection was applied using a *p*-value threshold for the partial t-statistic of 0.1. The threshold of 0.1 was chosen as opposed to the commonly applied threshold of 0.05 to minimize the risk of overlooking potentially critical process parameters. A strong heredity principle was followed i.e. if a two-factor interaction is included in the model, the main effects of both factors involved in the interaction are included in the model as well (even if the main effects are not significant with the chosen threshold). To ensure model adequacy, a thorough analysis of the model residuals is performed to check whether any of the assumptions for regression analysis are violated. i.e. the model errors are statistically independent, of constant variance, and normally distributed.

The unit operations were described by the specific clearance or yield for a given quality attribute. Clearances were used to describe the performance of the respective unit operation. Specific clearances are clearances calculated from impurity concentrations that are normalized to the amount of total product. This harbors the advantage that the values are independent of the scale and total volume. The specific clearances and yields were described by OLS models or observed mean values (in the absence of OLS models) with their respective calculated uncertainty. Models describing the specific clearance as a function of process parameters are termed “DoE Model” and were derived from small scale experiments. Models describing the specification clearance as function of the input material are termed “SC Model” and were derived from manufacturing data. If neither a DoE model nor a SC model was available, the specific clearances were described by fitting a normal distribution to the available manufacturing data.

If more than one OLS model was available for a unit operation, both models were used to describe the unit operation. As the specific impurity loading concentration was not included as a factor in the DoE, interaction effects between factors investigated in the DoE and the specific impurity loading concentration were assumed not to be expected.

The linkage of DoE models and specific clearance models was performed as described elsewhere (Zahel et al., 2017). The combination of DoE model and load model predictions was performed according to Eq. 3, where ${\hat{SC}}_{i}$ denotes the specific clearance predicted from DoE model, ${\hat{SC}}_{i}\left({PP}_{i}\right)$ denotes the specific clearance predicted from the process parameters, $\hat{SC}\left({SLC}_{i}\right)$ denotes the specific clearance predicted from the specific clearance model using the input concentration from the simulation ( ${SLC}_{i}$ ) and $\hat{SC}\left(\bar{{SLC}_{DoE}}\right)$ denotes the specific clearance predicted from the specific clearance model using the concentration of the starting material of the DoE ( $\bar{{SLC}_{DoE}}$ ). The runs of a DoE were performed with the same starting material. The DoE model is valid for the concentration of the starting material used in the DoE. Therefore, the change in specific clearance from the DoE start concentration to the simulation input concentration was used as correction factor.

$${\hat{SC}}_{i}=\hat{SC}\left({PP}_{i}\right)∙\frac{\hat{SC}\left({SLC}_{i}\right)}{\hat{SC}\left(\bar{{SLC}_{DoE}}\right)}$$

Eq. 3

### 2.5 Linkage of unit operations using the integrated process modelling technology

The IPM technology applied in this contribution is described in detail elsewhere (Zahel et al., 2017). The principle behind the IPM is to concatenate models describing the CQA values of individual unit operation together in order to predict the CQA distribution at each intermediate unit operation and ultimately at drug substance.

To account for error propagation during this concatenation, a Monte Carlo simulation is performed in the following way:

A pre-defined number of runs through 9 unit operations are simulated for each response, each using a set of different process parameter values drawn randomly from the their normal operating range represented by a normal distribution. Only set-point values of process parameters were available at the time of analysis. Without loss of generality of the approach, the coefficient of variation of each parameter was assumed to be 3%. The technical realization of the normal operating range is given in the results section.

**FIGURE 3:**
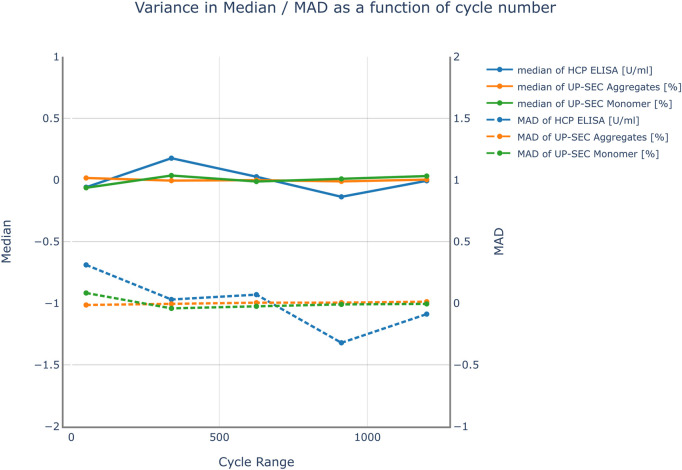
Variance of the median response and the mean absolute deviation (MAD) over the number of cycles used in the Monte Carlo simulation for all responses. The results were mean centered for visualization purposes. No severe changes in variance of the median prediction and the prediction variance were observed. Therefore, it was not expected that an increase in the number of simulation cycles improves the accuracy of the model predictions.

The impact of the number of Monte Carlo runs on the variance of the mean prediction and the prediction variance was investigated for all investigated responses. The results are shown in Figure 3. The impact of the number of simulation runs on the results was investigated in a range from 50 to 1200 simulations. No severe changes in variance of the median prediction and the prediction variance were observed. For that reason, 800 simulation runs were chosen for the subsequent parameter sensitivity analysis. This number leads to simulation cycles that can be conducted in a reasonable amount of time.

Unit operation performances are modelled as a function of process parameters (using OLS) and have some variance associated with them. Using this information, an uncertainty interval is defined around the mean prediction representing the uncertainty of the model prediction. Without loss of generality, 95% prediction intervals were chosen for the IPM. That is, for each simulated run, a response value is drawn randomly from this uncertainty interval around the mean.

Using the predicted clearance of a unit operation and the available load concentration, the pool concentration is calculated.

Special consideration is given to the simulated load values that fall outside the range of the observed load values used to train the model. Similar to Zahel et al., no extrapolation of clearances outside of the observed load models was performed (Zahel et al.). If simulated load values outside fall outside of the range, the clearance of the unit operation was assumed to be constant. For impurities (CQAs that need to be decreased), this approach might underestimate the clearance for load values higher than the observed range used to fit the models. This is considered more conservative from a risk based approach. For load values lower than the observed range the clearance might be overestimated. For setting up acceptance criteria the load values are gradually increased for each unit operation. For that reason, this case was not observed. For purities (CQAs that need to be increased) the signs need to be reversed.

The overall result of the Monte Carlo simulation with varying process parameters is a distribution for a specific CQA in the pool of the last unit operation, i.e. in drug substance. This distribution may be used to verify OOS event probabilities, given process parameter and model variability.

### 2.6 Calculation of OOS events

The number of out-of-specification events was calculated according to Taylor et al. (Taylor et al., 2021). A normal distribution was fit to the data. The OOS probability was defined by the area under the curve that lies beyond the drug substance specification limit. The parameters of the normal distribution were the arithmetic mean and the upper 80% confidence interval of the standard deviation. The upper confidence of the standard deviation was used to provide a fair comparison between the simulated runs and the real manufacturing runs, because of the large difference in sample sizes (800 simulated runs vs. 10 manufacturing runs).

## 3 Results

### 3.1 Description of the integrated process model

A pre-requisite of setting up an IPM is that the quality attributes to be modelled are measured both as input and output of the unit operations under investigation. Due to data availability, the IPM for HCP ELISA was set up from unit operation 1 to unit operation 6. For UP-SEC Aggregates and UP-SEC Monomer the integrated process model was set up from unit operation 2 to unit operation 9. *Table 2* outlines how the unit operations were modelled for each CQA.

**TABLE 2:** Summary of models used for modelling each unit operation and each CQA. Models describing the specific clearance as function of process parameters are termed “DoE Model”. Models describing the specification clearance as function of the input material are termed “SC Model”. If neither a functional relationship of specific clearance on process parameters nor on the input material was found, the unit operation was described by the specific clearance observed in manufacturing, termed “Manufacturing SC”.

	HCP ELISA	UP-SEC monomer	UP-SEC aggregates
HCCF			
Capture	DoE Model+SC Model	Manufacturing SC	Manufacturing SC
Virus Inactivation	Manufacturing SC		
Depth Filtration	SC Model	SC Model	SC Model
AEX	SC Model		SC Model
CEX	DoE Model	DoE Model+SC Model	DoE Model
Viral Filtration		Manufacturing SC	Manufacturing SC
HIC		SC Model	DoE Model
UFDF		Manufacturing SC	
Bulk		SC-Model	Manufacturing SC

### 3.2 Definition of the NOR

For modeling the process parameters, the definition for the NOR as outlined by FDA and EMA is followed.

“The NOR describes a region around the target operating conditions that contain common operational variability (variability that can’t always be controlled)” (EMA/213746/2017, o. J.).

For the purpose of the ensuing analysis, we aim to provide a technical realization of this definition. To our knowledge no mathematical description of the normal operating range has been given so far.

Without loss of generality, this operational variability is assumed to be caused by experimental errors stemming from several independent, uncontrollable sources. Therefore, it is sufficient to assume that continuous process parameter values follow a normal distribution (with the target operating value (set-point) being the most probable one (mean of the distribution)). This holds true for any targeted continuous process parameter value. For parameters that are controlled in such a way the NOR follows a normal distribution described by two parameters (mean and standard deviation). For parameters that don’t need to meet a target, but are allowed to stay within a range according to manufacturing batch records other distributions might be applicable (such as uniform distributions, poisson distributions or truncated normal distributions).

Each process parameter value has a certain probability of being observed associated with it; the set-point is the most probable value. The process parameter distribution follows a normal distribution around the set-point. The normal operating range (NOR) of a process parameter is then defined as the lower and upper boundary of the distribution covering a pre-defined area under the curve (e.g. +/- 3 standard deviations around the set-point). The values within the NOR are normally distributed (and not uniformly distributed). Following this definition, the normal operating range is a function of the applied set-points and is subject to change in the case the process parameter set-point is changed.

As a consequence, the results of the integrated process model are only valid if the process is controlled at target conditions including the uncertainty (NOR) around it, that is, whereat all PPs are kept at set-point and the process variability (i.e. standard deviation) does not increase.

### 3.3 Plausibility check of the integrated process model

Each individual OLS model was assessed individually based on model statistics R2, Q2, *p*-values, and RMSE as described in the material method section. The quality of the simulation with the concatenated models was assessed by comparing the predictions of the IPM with actually performed manufacturing runs at target conditions. Additionally, the predicted OOS rate was compared to the OOS rate calculated from the manufacturing runs.

**FIGURE 4:**
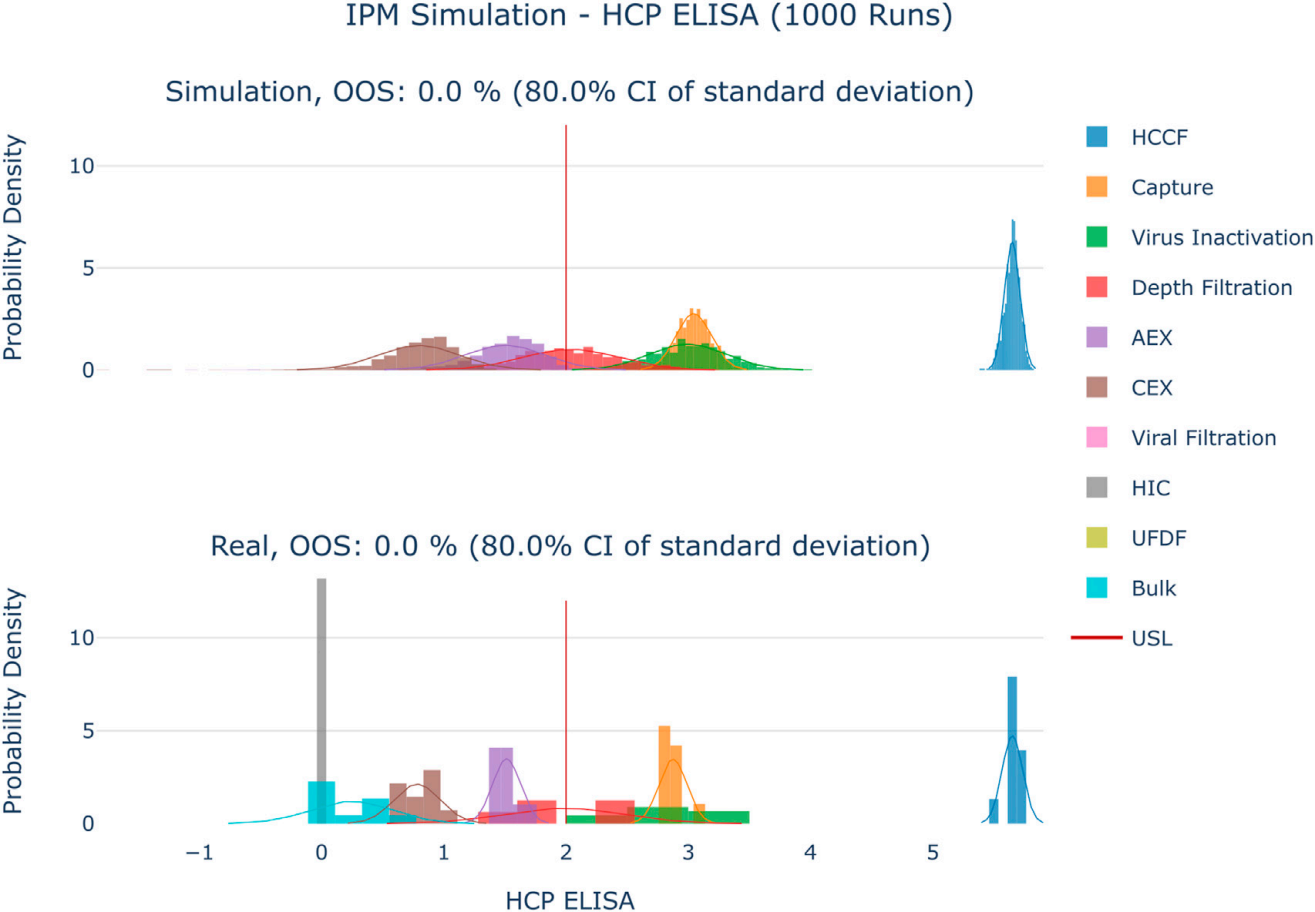
Comparison of distributions of the simulation to 10 large scale manufacturing runs for the CQA HCP ELISA. The upper plot shows the simulated data based on 1000 simulations performed at set-point conditions. The lower plot shows the data from 9 large scale runs. Due to the large value range of this CQA the values were logarithmically scaled for visualization purposes.

The results are shown in Figure 4–Figure 6. The span of the bar in the histograms was normalized in a way that the sum of all bin areas equals 1 (i.e., the area of each bar corresponds to the probability that an event falls into that bin). For that reason, the height of the bars (i.e., the probability densities) between the simulated values and the real data might differ, but the integrals equal 1. Therefore, the y values in these plots are not relevant for comparing the simulation with the real data. For all investigated CQAs, the simulated distributions fit quite well to the available manufacturing data. The predicted OOS probabilities (given in the plot titles) are in the same range as the OOS calculated from the manufacturing data. Based on these results the set-up model framework is regarded as fit for the application of setting up acceptance criteria.

**FIGURE 5:**
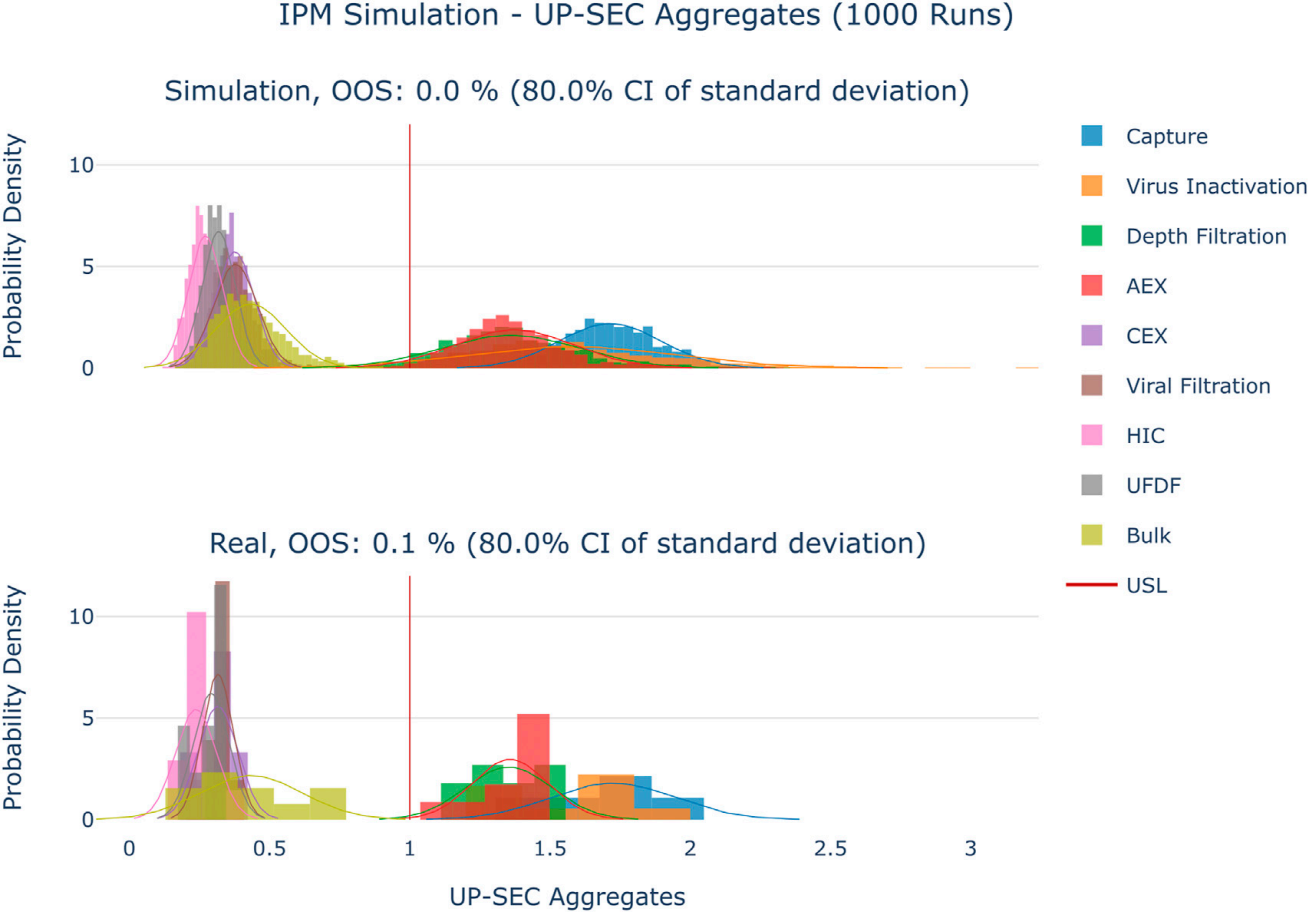
Comparison of distributions of the simulation to 10 large scale manufacturing runs for the CQA UP-SEC Aggregates. The upper plot shows the simulated data based on 1000 simulations performed at set-point conditions. The lower plot shows the data from 9 large scale runs.

**FIGURE 6:**
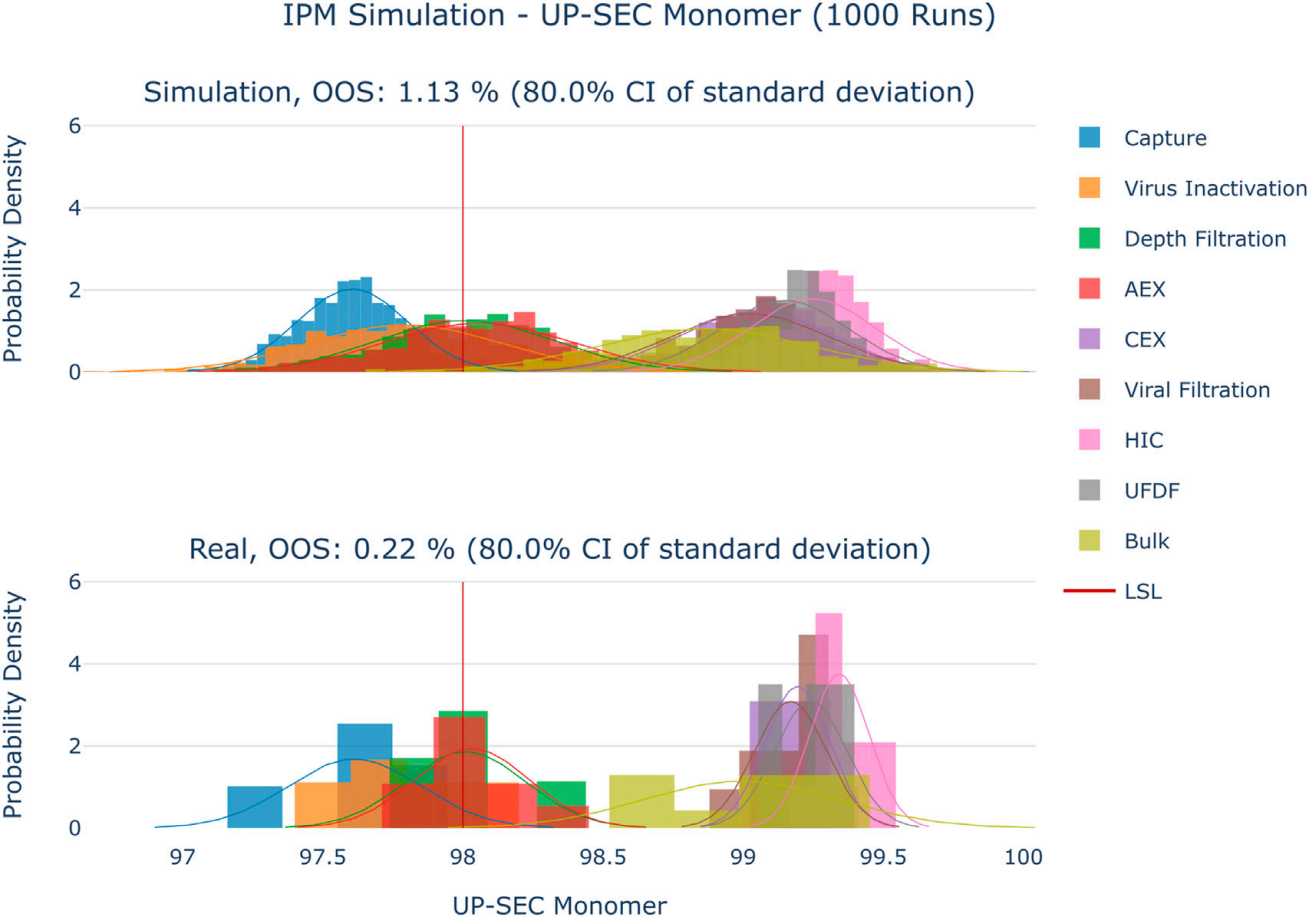
Comparison of distributions of the simulation to 10 large scale manufacturing runs for the CQA UP-SEC Monomer. The upper plot shows the simulated data based on 1000 simulations performed at set-point conditions. The lower plot shows the data from 9 large scale runs.

The definition of the Acceptance Criteria in ICH Q6B was followed. The statement “considered acceptable for it intended use” is interpreted in the following way: The drug substance material is considered acceptable for its intended use, if it conforms to the drug substance specification limits.

### 3.4 Definition and calculation of intermediate criteria

Following the outlined definition, the intermediate acceptance criteria will be defined by performing a parameter sensitivity analysis (PSA) within the IPM simulation framework. It will be assessed how a change in CQA load values in an intermediate unit operation affects out-of-specification (OOS) events at drug substance level.

For each CQA, the PSA was conducted as follows:

1) The screening range for the PSA was calculated from available manufacturing data. A range of plus/minus 10 standard deviations around the observed mean in the pool of the unit operation was calculated. The screening range was divided into 15 equidistant segments. If this resulted in negative values, the screening range was decreased by limiting it to positive values only.
2) The CQA’s pool value of the UO, for which the acceptance criteria are calculated, (= load value of the next UO) is set to a fixed value.
3) An IPM Monte Carlo simulation consisting of 800 simulated runs was performed according to the description in section 3.1.2, where all process parameters are randomly drawn from their normal operating range.
4) The number of OOS results for the CQA and a corresponding OOS probability is calculated.
5. Steps 2-5 are repeated for each of the screening range segments defined in step 1.
6) The intermediate acceptance criteria is then defined by the CQA pool concentration that results in the pre-defined OOS probability.

**FIGURE 7:**
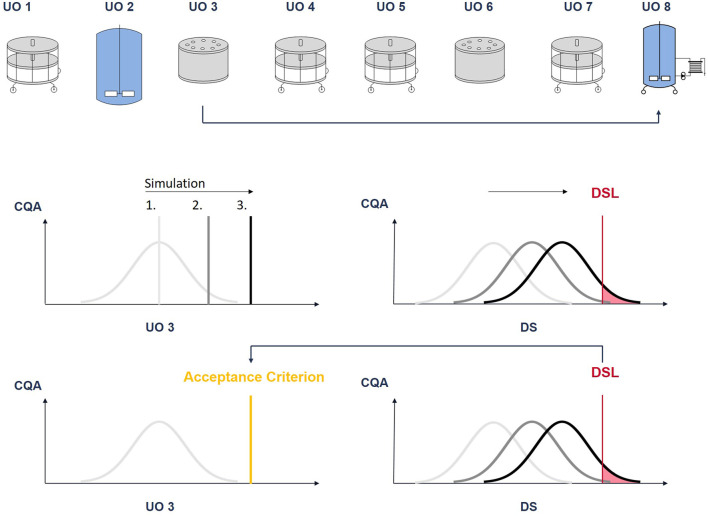
Illustration of the procedure to derive intermediate acceptance criteria: The CQA pool value at unit operation 3 is increased in discrete steps. At each step a Monte Carlo simulation is performed. With each step, the CQA distribution at DS moves towards the drug substance limit (DSL). The pool value, where a predefined fraction of the CQA distribution lies outside the DSL (out-of-specification probability), will define the iAC.

The procedure is then repeated for each CQA in each UO. An illustration of this procedure is given in Figure 7.

For the case study an OOS probability of 5% was defined as threshold.

### 3.5 Case study–Comparison of approaches for setting up acceptance criteria

**FIGURE 8:**
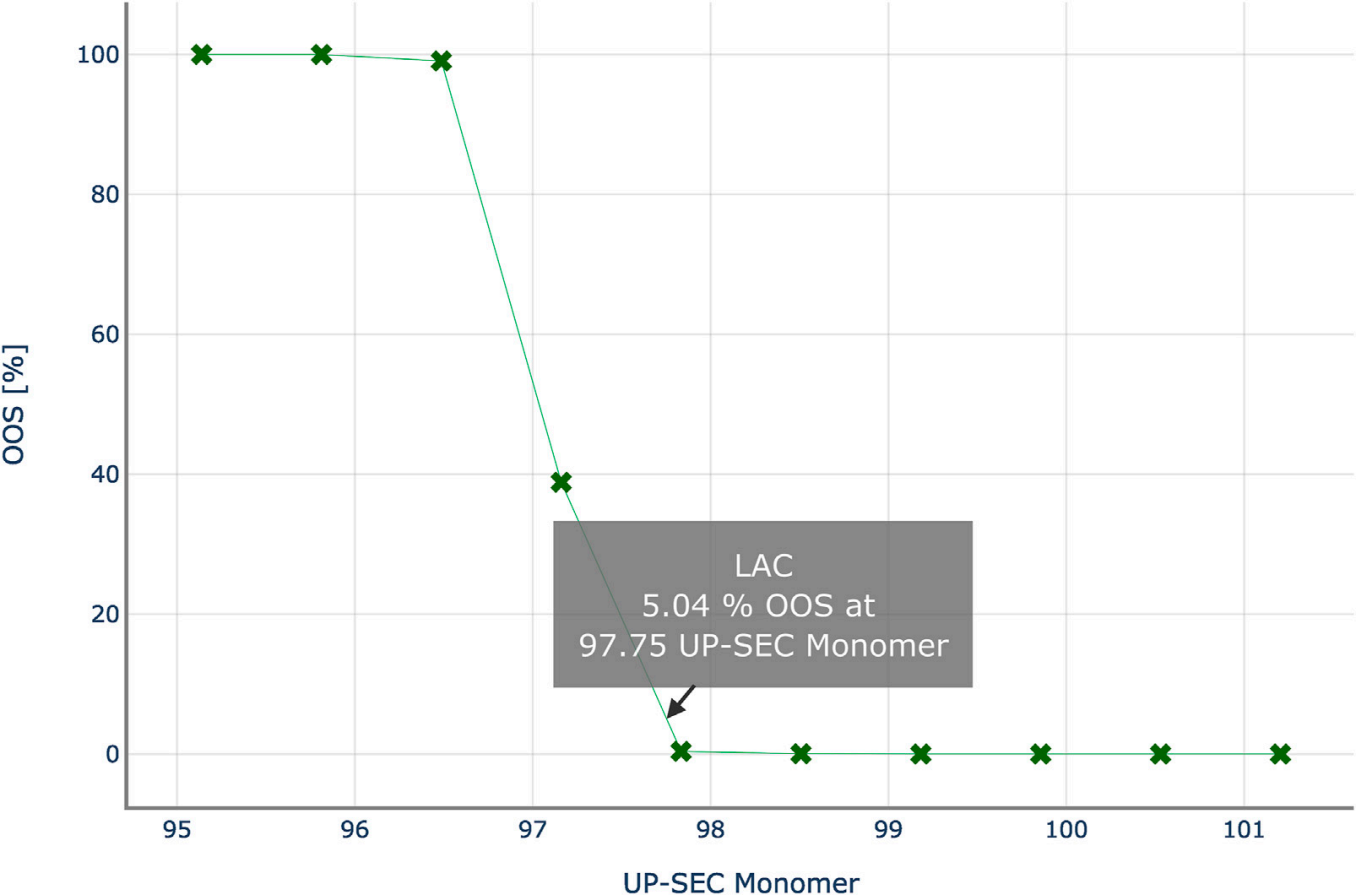
Out-of-specification probability at drug substance for various theoretical UP-SEC Monomer pool values in unit operation 2. Intermediate acceptance criteria are defined, as the CQA’s pool value, for which the out-of-specification probability equals a critical threshold, here 5%.

Figure 8 shows the results of the PSA to determine the intermediate acceptance criteria or UP-SEC Monomer in unit operation 2. For each data point, i.e. for a specific CQA pool value, CQA distributions at DS are predicted, and the probability to generate an out of-specification (OOS) limit is calculated. The OOS probability is then plotted as a function of the pool value. With each step the CQA distribution at drug substance moves towards the specification limit, increasing the risk of OOS events. At a 5% OOS probability, the proposed upper iAC for UP-SEC Monomer at unit operation 2 is 96.71% for the lower specification limit of 98%.

This procedure was followed for all unit operations and all CQAs under investigation. The corresponding plots are provided in the appendix.

In the case study the IPM derived acceptance criteria were compared to acceptance criteria based on + - three standard deviations. For all three responses only 1-sided specification criteria were defined. For this reason, the IPM derived acceptance criteria are also 1-sided. For impurities (HCP ELISA and UP-SEC Aggregates) an upper limit was defined and for purities (UP-SEC Monomer) a lower limit was defined.

**FIGURE 9:**
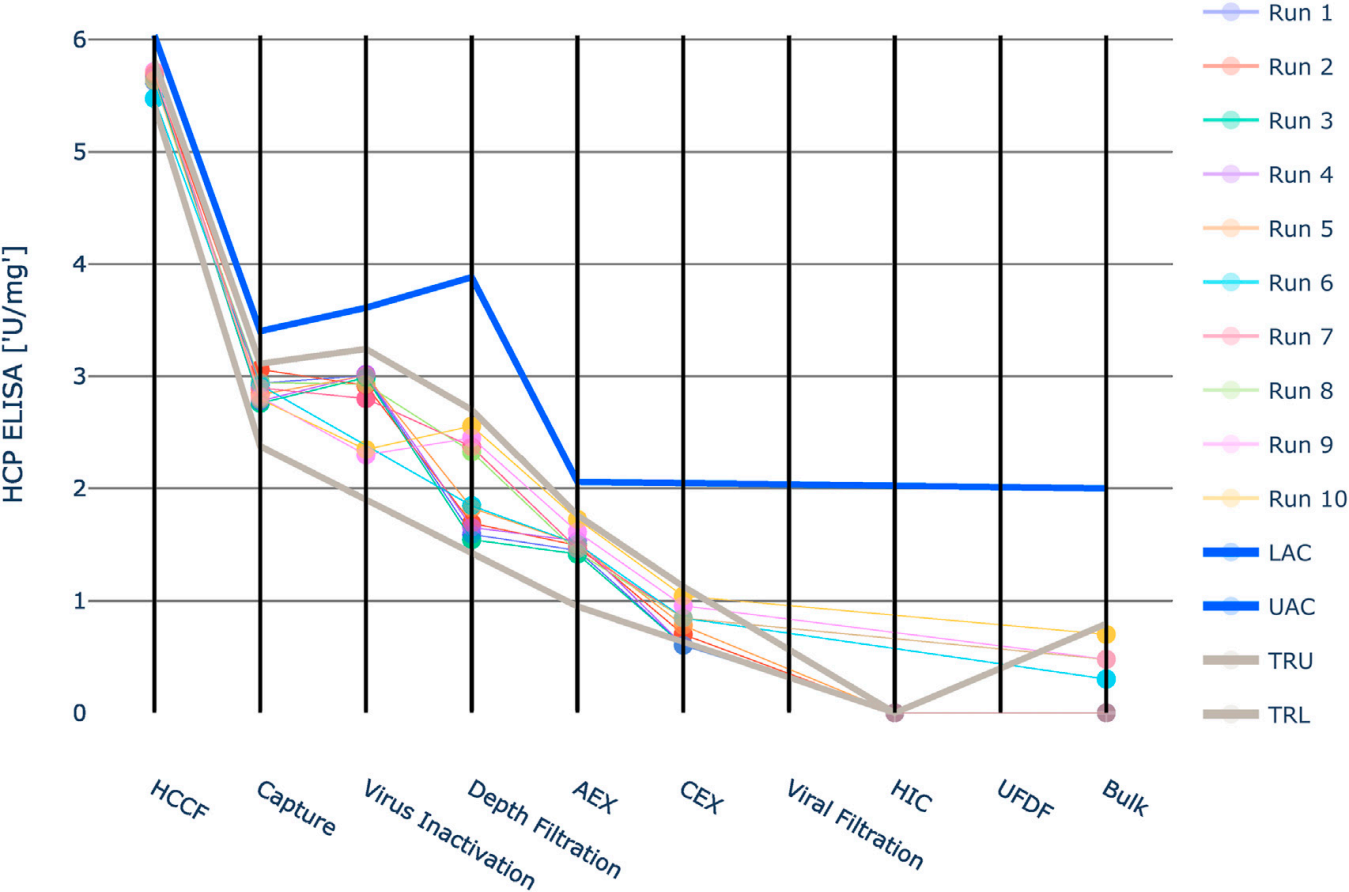
Graphical representation of the intermediate acceptance criteria (blue line) across the entire downstream process for the response HCP ELISA. Available large scale, manufacturing data per batch (circles) and three standard deviation ranges (grey lines) are given as well. The shown iACs at DS are the DS specification limits. Due to the large value range of this HCP ELISA the values were logarithmically scaled for visualization purposes. Except for unit operation 7 at the last five unit operations the intermediate acceptance criteria were set equal to the drug substance specification limits. At unit operation 7 CQA measurements are available, however they all represent one value: the limit of quantification. For that reason no standard deviation could be calculated and integrated process modeling could not be applied.

Due to the large value range of HCP ELISA, the values were logarithmically scaled for visualization purposes (Figure 9). For HCP ELISA, the IPM derived acceptance criteria were higher than the upper three standard deviation limits in all investigated unit operations. Especially in the first four unit operations the three standard deviation derived limits are much tighter than the IPM derived limits. Runs that fall outside the 3SD limit might still exhibit an acceptable out-of-specification probability. If these 3SD limits are applied, it might lead to the issue that alerts are raised unnecessarily. Except for unit operation 7 at the last five unit operations no data was available for HCP ELISA. At unit operation 7 CQA measurements are available, however they all represent one value: the limit of quantification. For that reason no standard deviation could be calculated and integrated process modeling could not be applied. The intermediate acceptance criteria were therefore set equal to the drug substance specification limits. This approach relies on the assumption that the impurity does not increase in these unit operations.

**FIGURE 10:**
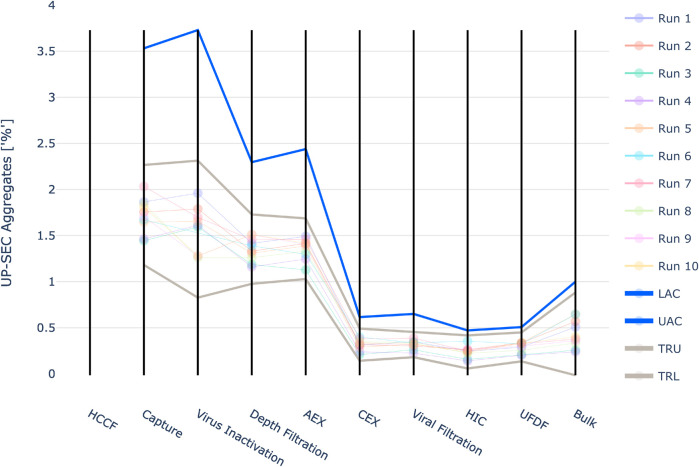
Graphical representation of the intermediate acceptance criteria (blue line) across the entire downstream process for the response UP-SEC Aggregates. Available large scale, manufacturing data per batch (circles) and three standard deviation derived ranges (grey lines) are given as well. The shown iACs at DS are the DS specification limits.

For UP-SEC Aggregates the IPM derived acceptance criteria were higher than the upper three standard deviation limits in all investigated unit operations (Figure 10). As described for the previous CQA if 3SD limits are applied, it might lead to the issue that alerts are raised unnecessarily.

**FIGURE 11:**
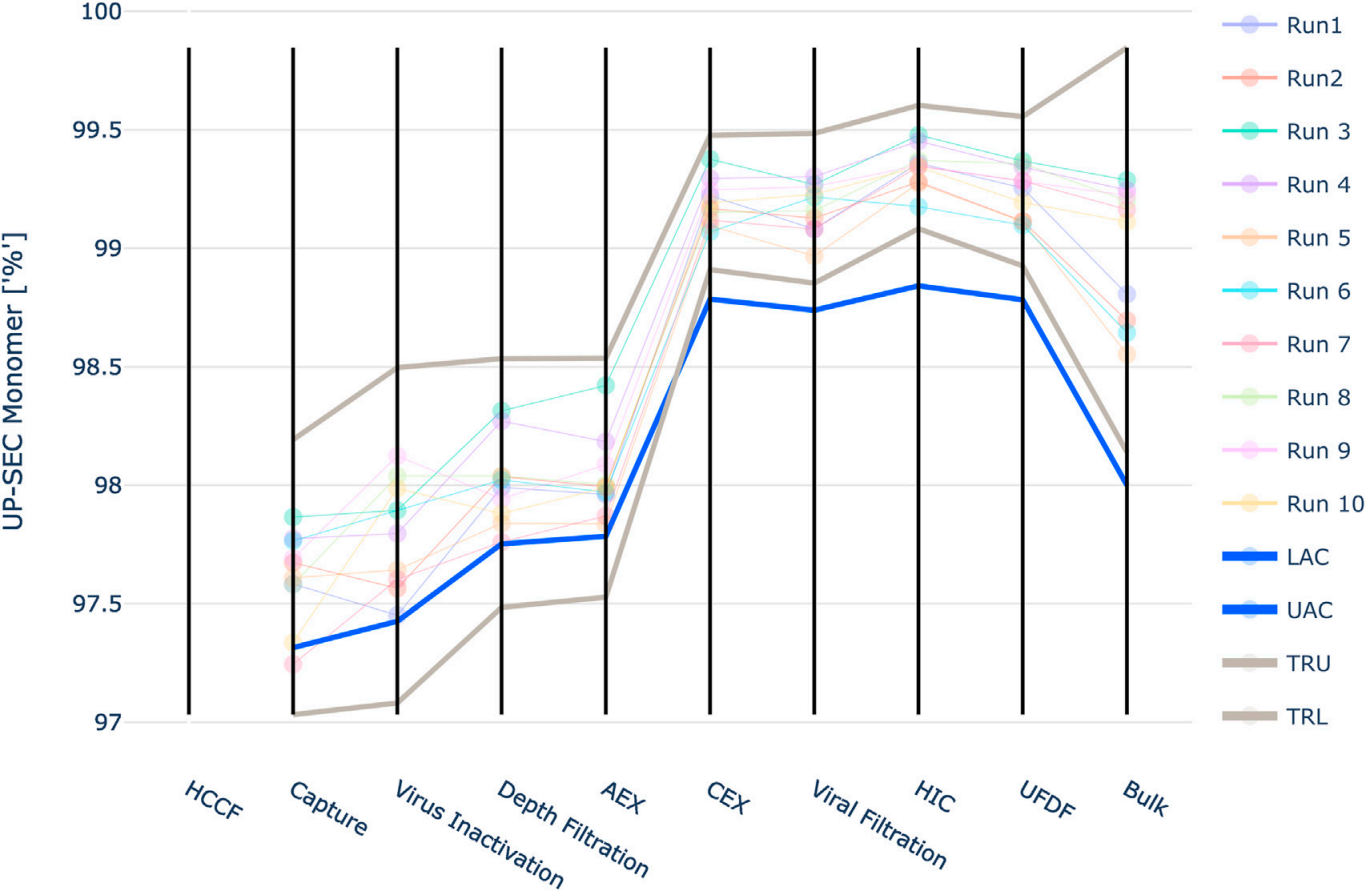
Graphical representation of the intermediate acceptance criteria (blue line) across the entire downstream process for the response UP-SEC Monomer. Available large scale, manufacturing data per batch (circles) and 3 standard deviation ranges (grey lines) are given as well. The shown iACs at DS are the DS specification limits.

For UP-SEC Monomer the IPM derived acceptance criteria lie close to the observed manufacturing values in unit operations 1 to 4 (Figure 11). For unit operation 2 a manufacturing run falls even below the acceptance criteria, although it still meets the final drug substance specification limit. The definition of the intermediate acceptance criteria is based on a probabilistic approach, i.e. at the intermediate acceptance criterion, there is a certain probability (here 5%) that the CQA does not meet drug substance specification limits. Consequently, even if a manufacturing run is close to the proposed intermediate acceptance criteria, this does not necessarily lead to the run being out of specification at DS. If it lies exactly at the intermediate acceptance limit, there is still a 95% probability that the run is within the specification limit.

Additionally, the lower limit of the three standard deviation derived ranges is lower than the IPM derived acceptance criteria for the first four unit operations. Based on the results of the IPM this means that the out of specification probability is larger than 5% at these limits. For unit operation 3 the lower 3 SD limit is 97.1%. At this value the IPM yields a 14.9% out of specification probability. If 100 runs were close to the lower 3 SD limit 14.9 runs would not meet the specification criteria at drug substance.

## 4 Discussion

Many contributions elaborate on methods to set up control strategies for process parameters (e.g. design space) (Abu-Absi et al., 2010; Jiang et al., 2010). A prerequisite for that is the knowledge, which levels of quality attributes are acceptable. Acceptance criteria serve as backbone for a proper control strategy on process parameters and material attributes. Often irrespective of the control strategy methodology, the reader is left alone in setting up acceptance criteria. Additionally EMA requires acceptance criteria for CPPs and CQAs to be part of the process validation protocol, which should be based on development data or documented process knowledge (European Commission 2015). However, here no specific guidance is provided to derive such limits.

Within this contribution we refined the definition of acceptance criteria by ICH Q6B by further specifying the term “for intended use” to having a link to final specification limits used for drug substance release (ICH Q6B, 1999). Additionally, we presented a methodology to calculate intermediate acceptance criteria based on drug substance specification limits and considers uncertainty around process parameters.

It should be emphasized that IPM derived acceptance criteria are only valid for a defined set of process parameter conditions. This means that if acceptance criteria were defined based on manufacturing runs at set-point conditions, they are only valid for runs that are performed at set-point. In the case of process changes, intermediate acceptance criteria need to be revised. This not only applies to the method presented in this contribution, but also applies to other approaches that rely on historic manufacturing data such as approaches that rely on min -max ranges. +/- 3 standard deviations, or statistical intervals (Seely et al., 2003; Orchard, 2006; Wang et al., 2007). Approaches that include data where variance was purposefully introduced into process parameters, as done in process development or process characterization studies, offer the advantage that the established models can easily be used to calculate acceptance criteria for the new process set-points without the need of acquiring new data (Montes, 2012; Burdick et al., 2017). Updating the acceptance criteria is in line with ICH Q8, which states that acceptance criteria can be updated in the case new process knowledge is available (ICH Q8 (R2), 2009, S. 8). Whereas ICH Q8 states that they should be updated in the case new process knowledge is available, we want to emphasize that they also need to be updated if process changes are implemented (e.g. process parameter set-points).

In this contribution we used OLS regression models to describe the individual unit operations. At the time of the case study the experimental work has already been conducted. The performed OFATs and DoEs were designed to be analyzed using OLS regression. This technique is the standard method for the analysis of DoEs. Care has to be taken, when extrapolation beyond the training range is performed. However, the described methodology for setting up acceptance criteria is not limited to OLS models. If mechanistic models are available model-based DoE approaches could be applied and the functional relationship between quality attributes and process parameters could be described by purely mechanistic or hybrid models (Kroll et al., 2017; Nold et al., 2021). For model-based approaches capturing the prediction uncertainty is not straight-forward and novel methods to do so are discussed in scientific literature (Briskot et al., 2019). However, an in-depth comparison of modelling approaches is beyond the scope of this contribution.

In addition to suitable data, the presented method requires knowledge in programming or scripting languages to concatenate the individual OLS models and perform the Monte Carlo simulations. In contrast, to that the 3SD approach can easily be applied in table calculation programs like MS Excel. Despite the complex knowledge required, we believe that the benefit of being able to leverage all available process knowledge in the form of statistical models in the integrated process model outweighs the increased analysis effort. Additionally, setting up integrated process models can be automated dependent on the digital maturity of the companies. If quality data and process parameter values are automatically collected in a centralized system the process of setting up an integrated process model can be facilitated.

The available guidelines encourage basing the definition of limits on the entirety of process knowledge. ICH Q6E states “In this respect, limits are justified based on critical information gained from the entire process spanning the period from early development through commercial scale production.” (ICH Q6B, 1999). ICH Q8 further emphasizes the fact that it should be justified how in-process controls contribute to the final product quality (ICH Q8 (R2), 2009, S. 8). ICH Q11 states that links between process and quality is needed (ICH, 2012, S. 11). The above approach puts the guidelines recommendations into practice. It combines the knowledge from small scale studies and manufacturing runs. Functional relationships of quality and process parameters are included. The results are based on drug substance specification criteria. Following the principle of the control strategy lifecycle as outlined in ICH Q8, acceptance criteria can be updated using the IPM as new knowledge is available (ICH Q8 (R2), 2009, S. 8).

The presented IPM approach models independently from each other. Hence, it relies on the assumption that there are no interactions between the studies quality attributes. This could be addressed by studying various CQA starting concentrations in wet-lab experiments and modelling CQAs as function of other CQAs. In that way multivariate range can be set up that not only consider multivariate dependencies on process parameters but also on other CQAs.

Currently most specifications are based on process variability and not patient-driven. We’d like to see future work that focuses on how to define drug substance/product specifications that are based on patient response (safety and efficacy). In order to achieve this, manufacturing data should be linked to data from the clinic. Additionally, the quantity and quality of the data is important.

**FIGURE 12:**
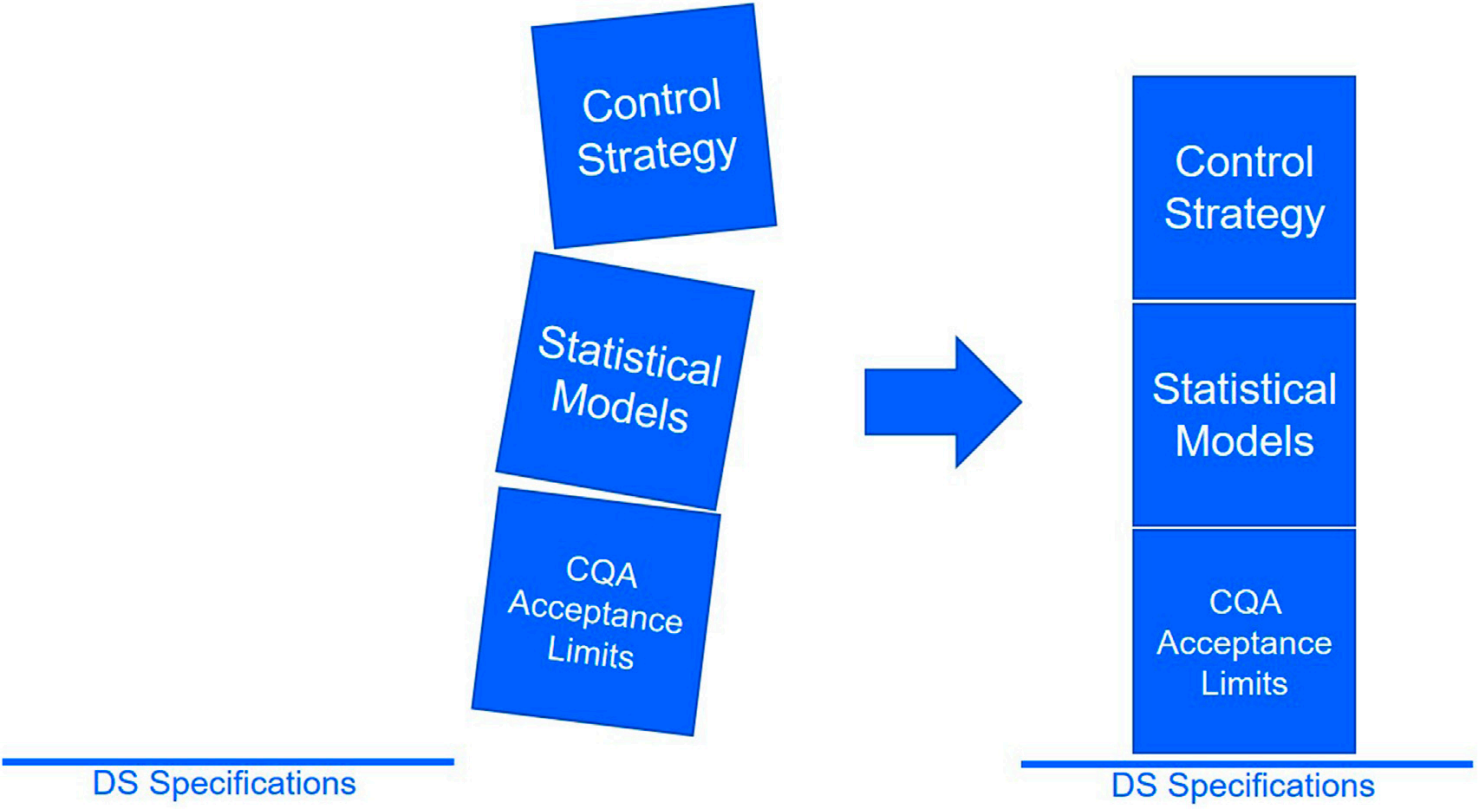
Specification-driven acceptance criteria provide a solid line of reasoning and enable robust control strategies for process parameters.

The aforementioned aspects of the IPM derived acceptance criteria provide a solid line of reasoning for justification in audits as they are built on the total amount of available evidence, while using already well established modelling techniques (i.e. OLS). The described methodology enables the definition of acceptance criteria based on the probability of reaching the specification limits. We therefore firmly support using specification-driven acceptance criteria form a solid base for activities in setting up control strategies (Figure 12). The IPM derived acceptance criteria may prove to be an excellent foundation for the establishment of patient centric specifications as correlations between product attributes and clinical outcomes are made.

## Data Availability

The datasets presented in this article are not readily available because the data used in this study was generated for a commercial manufacturing process. The rights to the dataset are owned by the company. Requests to access the datasets should be directed to lukas.marschall@koerber.com.
