# Supplementary material for: Specification-driven acceptance criteria for validation of biopharmaceutical processes
Source: Front Bioeng Biotechnol. 2022 Sep 23;10:1010583. doi: 10.3389/fbioe.2022.1010583 (PMC9537461; doi:10.3389/fbioe.2022.1010583)
Supplement: Supplementary file 1 [file DataSheet1.docx]

Supplementary Material

Table 1: Overview of models describing the specific clearance as a function of process parameters. R^2^, Q^2^, and p-values, as well as residual analysis, were used alongside process expertise to determine acceptance of the model within the IPM.

| **Unit Operation** | **CQA** | **Adj. R2** | **Q2** | **p-value (F-Statistic)** | **No of Obs** | **Parameters** |
| --- | --- | --- | --- | --- | --- | --- |
| Capture | HCP ELISA | 0.633 | 0.427 | 0.0674 | 5 | Const (-1.30e-17), Load Pool Temperature (8.51e-0.1) |
| CEX | HCP ELISA | 0.913 | 0.868 | 2.36E-05 | 11 | Const (-2.637e-16), Elution Buffer pH (-8.397e-01), Elution Buffer Cond (-4.745e-01) |
| CEX | UP-SEC Aggregates | 0.985 | 0.954 | 2.70E-07 | 11 | Const (-1.665e-16), Elution Buffer pH (-7.616e-01), Elution Buffer Cond (-5.822e-01), Elution Buffer pH*Elution Buffer Cond (-2.521e-01) |
| CEX | UP-SEC Monomer | 0.996 | 0.987 | 1.14E-06 | 11 | Const (0.246), Elution Buffer pH (-0.644), Elution Buffer Cond (-0.571), Elution Buffer pH*Elution Buffer Cond (-0.444), Elution Buffer pH^2 (-0.179), Elution Buffer Cond^2 (-0.092) |
| HIC | UP-SEC Aggregates | 0.453 | 0.321 | 0.00572 | 17 | Const (-7.494e-16), Loading Pool pH (-5.001e-01), Loading Pool Temp (5.188e-01) |

Table 2: Overview of models describing the specification clearance as function of the input material. R^2^, Q^2^, and p-values, as well as residual analysis, were used alongside process expertise to determine acceptance of the model within the IPM.

| **Unit Operation** | **CQA** | **Adj. R2** | **Q2** | **p-value (F-Statistic)** | **No of Obs** | **Parameters** |
| --- | --- | --- | --- | --- | --- | --- |
| Capture | HCP ELISA | 0.424 | 0.427 | 0.0246 | 10 | Const (4.921), HCP ELISA (0.000003) |
| Depth Filtration | HCP ELISA | 0.892 | 0.869 | 7.78E-05 | 9 | Const (-1.429), HCP ELISA (0.00438) |
| Depth Filtration | UP-SEC Aggregates | 0.706 | 0.565 | 0.0028 | 9 | Const (-0.973), UP-SEC Aggregate (0.712) |
| Depth Filtration | UP-SEC Monomer | 0.487 | 0.352 | 0.0219 | 9 | Const (-0.857), UP-SEC Monomer (-0.00874) |
| AEX | HCP ELISA | 0.835 | 0.691 | 0.000136 | 10 | Const (0.233), HCP ELISA (0.00614) |
| AEX | UP-SEC Aggregates | 0.699 | 0.65 | 0.000119 | 14 | Const (-0.368), UP-SEC Aggregate (0.268) |
| CEX | UP-SEC Monomer | 0.845 | 0.812 | 2.10E-06 | 14 | Const (0.358), UP-SEC Monomer (-0.00353) |
| HIC | UP-SEC Monomer | 0.362 | 0.205 | 0.0387 | 10 | Const (0.651), UP-SEC Monomer (-0.00655) |
| Bulk | UP-SEC Monomer | 0.472 | 0.36 | 0.00397 | 14 | Const (-1.033), UP-SEC Monomer (0.0104) |

Table 3: Specific clearances were calculated by fitting a normal distribution to the available manufacturing data.

| **Unit Operation** | **CQA** | **Mean** | **Std** | **No of Obs** |
| --- | --- | --- | --- | --- |
| Virus Inactivation | HCP ELISA | 0.131 | 0.636 | 9 |
| Virus Inactivation | UP-SEC Aggregates | 0.103 | 0.196 | 9 |
| Virus Inactivation | UP-SEC Monomer | 0.00198 | 0.0029 | 9 |
| Viral Filtration | UP-SEC Aggregates | -0.00243 | 0.0932 | 14 |
| Viral Filtration | UP-SEC Monomer | -0.000249 | 0.000769 | 14 |
| UFDF | UP-SEC Monomer | -0.00103 | 0.000491 | 10 |
| Bulk | UP-SEC Aggregates | -0.282 | 0.223 | 14 |
